# Reaffirming the role of iodine in cognitive health: a call for lifespan-based public health action

**DOI:** 10.1097/MS9.0000000000004215

**Published:** 2025-10-28

**Authors:** Zehra Sadia, Tooba Khurshid, Musawer Khan, Kamil Ahmad Kamil

**Affiliations:** a Internal Medicine Department, Karachi Medical and Dental College, Karachi, Pakistan; b Internal Medicine Department, Combined Military Hospital Quetta, Baluchistan, Pakistan; c Internal Medicine Department, Mirwais Regional Hospital, Kandahar, Afghanistan

**Keywords:** cognitive health, early childhood, iodine deficiency disorder, neurodevelopment, pregnancy

## Abstract

Iodine deficiency critically affects cognitive health across the lifespan, particularly during pregnancy, early childhood, and older age. This editorial highlights the evidence linking deficiency to impaired neurodevelopment and accelerated cognitive decline. Despite effective interventions such as salt iodization, public awareness remains low. We urgently call for integrated, lifespan-based public health strategies – combining strengthened policies, targeted education, and further research – to ensure iodine sufficiency and to protect global cognitive well-being.

*To the Editor,*

Iodine is a vital micronutrient essential for thyroid function, neurodevelopment, and overall metabolic regulation throughout human lifespan. It plays a critical role in the development of the central nervous system and supports thermoregulation, growth, and reproductive functions^[1,2]^. For adults, the recommended daily intake is 150 µg, increasing to 200–250 µg during pregnancy and lactation, whereas infants require approximately 40 µg per day^[3]^.

Iodine deficiency remains a significant global public health concern, contributing to a range of intellectual, metabolic, and thyroid-related disorders^[4]^. The impact varies across age groups, with pregnant women and young children being particularly vulnerable to iodine’s crucial role in neurodevelopment. A randomized controlled trial in Ethiopia demonstrated that preconception iodized salt consumption significantly improved cognitive outcomes in children^[5]^. Severe iodine deficiency during pregnancy and infancy can result in miscarriage, stillbirth, neonatal hypothyroidism, cretinism, growth retardation, and increased infant mortality with insufficient iodine status reported in 16.1–84.0% of pregnant women across studies^[6]^.

Despite these risks, the awareness of the importance of iodine remains low in certain populations. For example, women of reproductive age in the UK often lack sufficient knowledge about iodine’s dietary sources and its role in pregnancy, leading to inadequate intake,only 39% were aware that iodine requirement increase in pregnancy and 71% reported using iodine-containing supplements^[7]^. A recent longitudinal study from Bangladesh further emphasized the value of urinary iodine concentration (UIC) as a biomarker for iodine intake, demonstrating its predictive power for cognitive development at ages 5 and 10 in children with 4%of having UIC <100 μg/L^[8]^.

Notably, iodine’s significance extends beyond early life. A study from China found an inverse relationship between iodized salt intake and cognitive decline in older adults, underlining the value of iodine in cognitive health in aging populations among 10 217 participants, 16.1% had cognitive impairments, iodized salt intake was associated with reduced odd of impairment (OR = 0.41,95% CI 0.35–0.48)^[9]^.

These findings suggest that iodine sufficiency should be maintained across all stages of life, especially in regions with suboptimal iodine intake.

Public health initiatives should prioritize awareness campaigns regarding the role of iodine in cognitive development, particularly during pregnancy and early childhood. Engaging local media within national strategies can promote the use of iodized salt and help prevent iodine deficiency disorders^[10]^. More robust public health efforts and research are needed globally, especially in countries such as China – to ensure adequate iodine intake across age groups.

Although an estimated 2 billion people suffer from iodine deficiency disorders worldwide, their specific biological mechanisms remain underexplored. Additional research is necessary to understand how iodine influences cognitive and metabolic pathways, which could lead to more targeted interventions^[11]^.

In summary, the consequences of iodine deficiency are profound, particularly for pregnant women, infants, and elderly individual. Addressing this global health issue requires a combination of policy-driven salt iodization programmes, early detection strategies, and widespread educational initiatives. A comprehensive, lifespan-based approach is essential for promoting cognitive well-being and prevents iodine deficiency across generations.

This editorial adheres to the **TITAN Guidelines 2025 on transparency, integrity, and the use of AI in medical writing and publishing**^[12]^.

## Data Availability

Not applicable.
